# FragVLib a free database mining software for generating "Fragment-based Virtual Library" using pocket similarity search of ligand-receptor complexes

**DOI:** 10.1186/1758-2946-4-18

**Published:** 2012-08-21

**Authors:** Raed Khashan

**Affiliations:** 1 Department of Pharmaceutical Sciences, College of Clinical Pharmacy, King Faisal University, Al-Ahsa, 31982, KSA, Saudi Arabia

## Abstract

**Background:**

With the exponential increase in the number of available ligand-receptor complexes, researchers are becoming more dedicated to mine these complexes to facilitate the drug design and development process. Therefore, we present FragVLib, free software which is developed as a tool for performing similarity search across database(s) of ligand-receptor complexes for identifying binding pockets which are similar to that of a target receptor.

**Results:**

The search is based on 3D-geometric and chemical similarity of the atoms forming the binding pocket. For each match identified, the ligand's fragment(s) corresponding to that binding pocket are extracted, thus, forming a virtual library of fragments (FragVLib) that is useful for structure-based drug design.

**Conclusions:**

An efficient algorithm is implemented in FragVLib to facilitate the pocket similarity search. The resulting fragments can be used for structure-based drug design tools such as Fragment-Based Lead Discovery (FBLD). They can also be used for finding bioisosteres and as an idea generator.

## Background

Due to the exponential increase of available ligand-receptor complexes, an increasing interest is dedicated to the development of computational tools for mining useful information which can facilitate the drug design and development process [1-4]. Therefore, we present a tool that mine database(s) of ligand-receptor complexes and generate a library of fragments for a target receptor so it can be used for structure-based drug design, such as Fragment-Based Lead Discovery (FBLD). FBLD is a computational approach which begins with a small low-affinity fragment(s) which bind to the target of interest, followed by a careful construction and optimization of these fragments to end up with a high affinity lead drug. In theory, this is a highly efficient approach for drug discovery, and it has become enormously popular in the past few years [1-4].

Our method relies on the graph representation of interfacial atoms for the ligand-receptor complex. Interfacial atoms are defined as nodes, and the distances between them are represented by edges connecting these nodes. Therefore, given a target receptor, we can perform a pocket similarity search by doing a ‘graph’ match. The match takes into account the chemistry and the 3D geometry of the atoms involved. For each match found, the ligand's atoms bound to the matched pocket are copied to the pocket of the target receptor and can, in theory, be regarded as binding fragments, since a similar pocket is shared. Performing this pocket similarity search over a dataset (or database) of receptor-ligand complexes will result into a virtual library of fragments (FragVLib).

Once the library of fragments is generated for the pocket of the target receptor, lead development can begin using three possible scenarios: Growing from these 'needles' into the depth of the pocket; linkage of two or more fragments into one compound with optimized potency; or merging two or more fragments in regions of mutual overlap [5].

### Implementation

**Figure 1:**
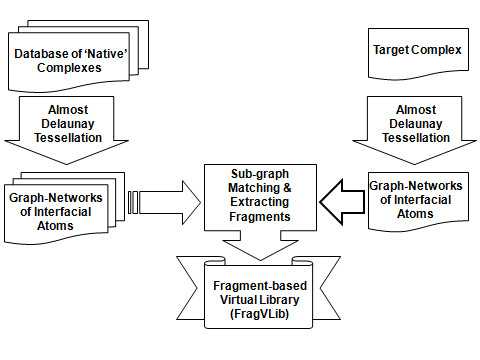
**Workflow of the program.** Interfacial graph are generated for both the target receptor and each receptor in the database under search. A pocket similarity match is performed, based on which, ligands’ fragments corresponding to the matched pockets are extracted.

**Figure 2:**
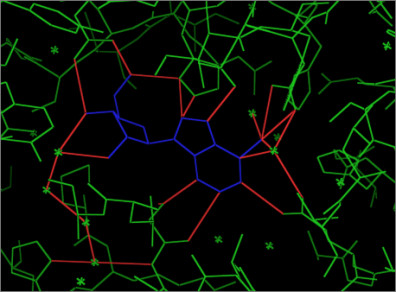
**An example of an interfacial graph for “adenosine deaminase” with PDB code “1a4m”.** The protein is shown in Green, the ligand in Blue, and the edges (in Red) are connecting interacting (interfacial) atoms within a distance cutoff 3.15 Ǻ. The interfacial graph is composed of the interfacial atoms the edges (in Red) which are connecting these atoms. Notice that if water molecules are not eliminated, they will be part of the graph forming a bridge between the ligand and protein interfacial atoms.

Starting with a target receptor (as a search query) and a database of 'native' ligand-receptor complexes (e.g., PDBbind [6,7]), we can begin the pocket similarity search. Notice that for the target receptor, a bound ligand or a user defined ligand is required to aid in identifying the binding pocket. The following paragraphs (along with Figure 1) explain the steps implemented in the program to use graph representation of ligand-receptor complexes, perform the pocket similarity search, and generate the fragment-based virtual library (FragVLib):

1. Identifying the interfacial atoms for the target complex and each 'native' complex in the database under search. Interfacial atoms are constituted by both the receptor and ligand atoms which are within certain cutoff distance. We use Almost-Delaunay (AD) tessellation [8] to perform this task; a unique advantage of AD tessellation is that it incorporates the imprecision of the point coordinates in defining the tessellation patterns. A threshold value (epsilon) is used to signify the minimum perturbation needed for an atom to be part of the interfacial graph. This is important when dealing with bad resolution receptor-ligand complexes. Figure 2 show an example of interfacial graph generated using AD tessellation.

2. Representation of interfacial atoms by un-directional graph; atoms are represented by labeled nodes, and distances connecting them are represented by labeled edges. Although not shown in Figure 2, atoms that are adjacent (covalently bound) to the interfacial atoms are also included in the graph. These adjacent atoms are also represented by labeled nodes, and the covalent bonds connecting them to interfacial atoms are represented as labeled edges. This will give a better chemical description of the interfacial atoms, plus, it is useful in identifying fragments as we will see in later steps. Table 1 shows the atom and bond types used to label the interfacial graphs.

**Table 1:** The list of atom and bond types used in labeling interfacial graphs

**Atom**	**Description**
C.3	Carbon sp3
C.2	Carbon sp2
C.1	Carbon sp
C.ar	Carbon aromatic
C.cat	Carbocation
N.3	Nitrogen sp3
N.2	Nitrogen sp2
N.1	Nitrogen sp
N.ar	Nitrogen aromatic
N.am	Nitrogen amide
N.pl3	Nitrogen trigonal planar
N.4	Nitrogen sp3 positively charged
O.3	Oxygen sp3
O.2	Oxygen sp2
O.co2	Oxygen in carboxylates and phosphates
S.3	Sulfur sp3
S.2	Sulfur sp2
S.O	Sulfoxide Sulfur
S.O2	Sulfone Sulfur
P.3	Phosphorous sp3
F	Fluorine
Cl	Chlorine
Br	Bromine
I	Iodine
Li	Lithium
Na	Sodium
Mg	Magnesium
Al	Aluminum
Si	Silicon
K	Potassium
Ca	Calcium
Cr.th	Chromium (tetrahedral)
Cr.oh	Chromium (octahedral)
Mn	Manganese
Fe	Iron
Co.oh	Cobalt (octahedral)
Cu	Copper
Se	Selenium
Mo	Molybdenum
Sn	Tin
Zn	Zinc
**Bond**	**Description**
1	Single bond
2	Double bond
3	Triple bond
am	Amide
ar	Aromatic
nc	Not connected

An option for labeling each atom type with the number of hydrogen atoms is available. This way, an sp3 Oxygen with one hydrogen atom is distinguished from an sp3 Oxygen with no hydrogen atoms; this is useful for hydrogen-bonding interactions.

**Figure 3:**
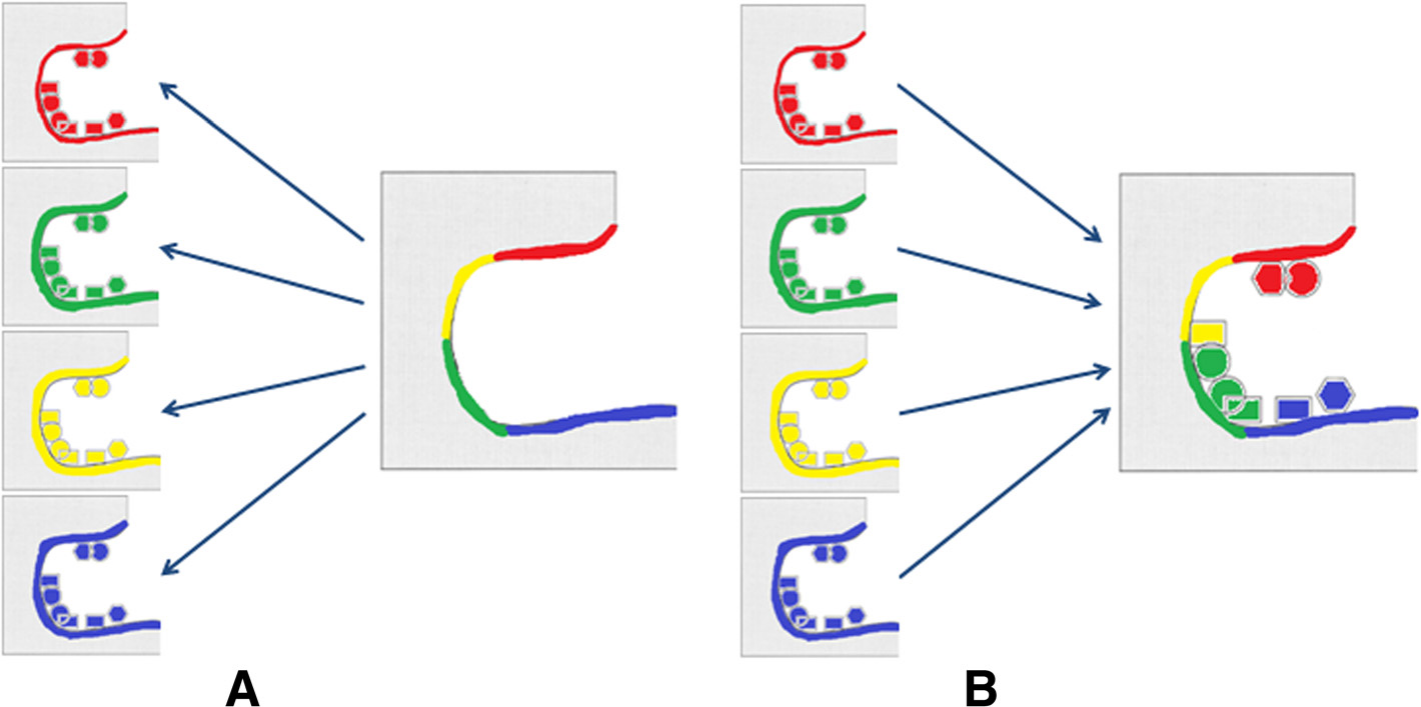
**Cartoon representation explaining the concept of generating the fragments.** A pocket similarity search is performed first (**A**), followed by extracting ligand's fragments (**B**).

3. Now since we have the complexes' interfaces represented by graphs, an efficient sub-graph match (we will address in the Discussion section later) can be performed between the interfacial graph of the target complex and the interfacial graph of each 'native' complex in the database under search. The match considers all possible sub-graphs and is performed over the nodes and edges composing the receptor side only, see Figure 3A. The match takes into consideration the labeling of the nodes and edges, and the 3D geometry of these nodes and edges. The size of an accepted sub-graph match (i.e., number of nodes in the sub-graph matched) is provided by the user as a range of a minimum and a maximum value. Also, for each sub-graph match identified, the 3D geometry is checked to make sure nodes super-impose within a user defined RMSD cutoff before it is accepted.

4. Once an accepted sub-graph match is found, the ligand's side nodes and edges in direct contact with the matched sub-graph are copied into the target receptor. This also includes the adjacent nodes and edges discussed in step 2. These nodes and edges form the fragment(s) obtained for this particular search, see Figure 3B. Copied nodes which are in collision (within a user-defined safety distance) with the target receptor's nodes are removed.

**Figure 4:**
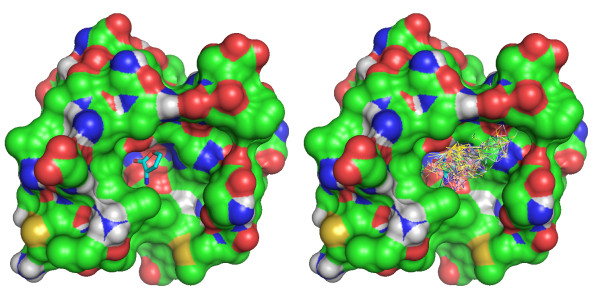
**An example of a library of 78 fragments generated by the program.** The picture to the left shows the target receptor with its ligand, and the one to the right shows the target receptor with the fragments generated. The target receptor is the protein-ligand complex with PDB code “2br6”, and the dataset used to perform the pocket similarity search is the refined set of PDBbind (v 2008) which contains 1401 complexes. The following parameters were used: Water molecules are removed; AD distance cutoff 5.8 Å with an epsilon value 0.01 Å; size we used for an acceptable sub-graph match is 8 atoms; maximum RMSD cutoff used is 0.1 Å; and the safety distance used to avoid a collision is 1.95 Å.

As a result of searching all 'native' complexes in the database, a collection of fragments (which we call FragVLib) filling the target receptor's pocket will be generated: The program provides all fragments from all matches copied into the binding pocket of the target receptor, see example in Figure 4. The name of the complex where each fragment came from is also provided. The user can explore each matching fragment alone to perform a growth into the binding pocket, or in combination with other fragments for careful linking or merging to construct the lead compound.

Notice that although the process involves many input parameters that are available to the user to explore, a default value is always provided for all these parameters, these default values are reported in the legend of Figure 4. Notice also that although it is recommended to have a ligand bound to the target receptor to define the binding pocket, the search can still be performed without the ligand as long as the binding pocket is known. In that case, a user-defined ligand (pseudo-ligand) can be designed by the user to help identifying the binding pocket. The user might be interested only in a part of the receptor’s binding pocket; therefore, a pseudo-ligand that is in contact only with that specific part can be designed for that purpose. Also, the distance cutoff which is used to identify the interfacial atoms (using AD tessellation) can be modified by the user. This is because some interactions occur over short distances, and others occur over long distances, the user might be interested in looking only at interactions occurring within certain distance cutoff. Water molecules can also be included as part of the interface or they can be omitted, which is the default option.

## Discussion

The program utilizes efficient tools for representing the interfacial atoms of the receptor-ligand complexes, as well as performing the pocket similarity search. However, the major drawback for the method is the fact that it relies on sub-graph matching as a way of performing the match searching process. Sub-graph mining in the presence of isomorphism is a well known NP-Complete problem [9] in the field of computer science. Such kind of problems is typically solved using techniques such as: Approximation, Randomization, Parameterization, Restriction, and Heuristic algorithms. Although these algorithms do not resolve the problem, yet, they give rise to substantially faster approaches in solving NP-Complete problems [10].

The algorithm used in our approach has a complexity in the order of O (P (log N) ^L^), where P, is the number of solutions, N is the size of the interfacial-graph, and L is the size of the match; to speed up the searching process, we implemented parameterization, restriction and heuristic algorithms. Parameterization is possible by fixing certain input parameters. For example, using short cutoff distances (default is 5.8 Å) in identifying interfacial atoms will result in interfacial-graphs that are smaller in size, and therefore, faster search is obtained. Short cutoff distances can be used when the target receptor’s binding pocket is expected to have interactions such as: hydrogen-bond, and ion exchange, which occur over short distances. If we expect hydrophobic interactions, which can occur over large distances, higher cutoff values can be used. Another parameter to consider is the size of the binding pocket at the target receptor; searches are usually performed to find fragments that can bind to a small particular area of the binding site at the target receptor, in that case, a user-defined ligand is needed for that purpose.

On the other hand, an example of restriction as a way of speeding up the searching algorithm is the use of an RMSD cutoff value for accepting the matched (super-imposed) interfacial-graphs, and the use of minimum and maximum values for an accepted sub-graph match. Finally, heuristic algorithms are also implemented in our searching technique; an example is the use of a canonical description of a graph or sub-graph, which uniquely identifies it. Using canonical description along with a specific way of growing the sub-graphs (a stepwise extension restricted to connected sub-graphs) provides a powerful technique to avoid redundant search, which is a core problem in sub-graph matching [11].

Aside from the limitations due to sub-graph mining complexity, the approach has a great advantage; it implicitly takes into account multi-body interactions (i.e., many bodies in contact [interacting] with each other) rather than pair-wise interactions. In other words, multiple chemical fragments (found in both ligand and receptor sides) are involved in the interfacial-graph matching search. This means that the search can result in multiple chemical fragments in the ligand side interacting with multiple chemical fragments in the receptor site cooperatively. This is important since cooperative interactions of multiple chemical fragments are hard to predict when designing a fragment-based lead compound.

## Conclusions

We present a program for database mining of the exponentially increasing number of receptor-ligand complexes, in particular, performing a pocket similarity search and extracting meaningful fragments that can be useful for Fragment-Based Lead Discovery. The program provides a very useful tool to explore available databases; it can function not only as a tool to provide virtual library of fragments for lead design, but also to aid in lead optimization (by providing bioisosteres for replacement), and as an idea generator.

## Availability

The program is written in C++, and it is publicly available freeware; it can be copied and distributed freely. The user manual and the pre-compiled executables (with default values) can be downloaded from "http://www.unc.edu/~raed/FragVLib.zip". It is easy to install (no external libraries) and easy to use. The development of FragVLib, in particular, the sub-graph matching algorithm, is an ongoing process: New features will be implemented from time to time.

## Competing interest

The author declares that he has no competing interests.
